# Licuri Oil (*Syagrus coronata*) as a Natural Oily Core for Cationic Polymeric Nanocapsules for Topical Formulation: Development, Characterization, and Incorporation into Hydrogels

**DOI:** 10.3390/molecules31173022

**Published:** 2026-08-28

**Authors:** Daniela Lana Tommasi Schmitt, Scheila Lopes dos Santos, Mariana Brunetto Büttenbender, Joice Maria Scheibel, Roberta Cougo Riéffel, Irene Clemes Kulkamp Guerreiro, Rosane Michele Duarte Soares, Alexandre José Macedo, Helder Ferreira Teixeira, Márcia Vanusa Silva, Maria Tereza dos Santos Correia, Karina Paese

**Affiliations:** 1 Programa de Pós-Graduação em Ciências Farmacêuticas, Faculdade de Farmácia, Universidade Federal do Rio Grande do Sul, Porto Alegre 90610-000, Brazil; 2 Laboratório de Nanocarreadores e Impressão 3D em Tecnologia Farmacêutica (Nano3D), Faculdade de Farmácia, Universidade Federal do Rio Grande do Sul, Porto Alegre 90610-000, Brazil; 3 Faculdade de Farmácia, Universidade Federal do Rio Grande do Sul, Porto Alegre 90610-000, Brazil; 4 Laboratório de Biomateriais Poliméricos (Poli-Bio), Universidade Federal do Rio Grande do Sul, Porto Alegre 91501-970, Brazil; 5 Laboratório de Biofilmes e Diversidade Microbiana, Centro de Biotecnologia e Faculdade de Farmácia, Universidade Federal do Rio Grande do Sul, Porto Alegre 91501-970, Brazil; 6 Departamento de Bioquímica, Centro de Biociências, Universidade Federal de Pernambuco, Cidade Universitária, Recife 50670-901, Brazil

**Keywords:** photoaging, sunscreen, avobenzone, *Syagrus coronata*, polymeric nanocapsules, photostability, hydrogel

## Abstract

Solar ultraviolet (UV) radiation is a major contributor to skin damage, making the regular use of broad-spectrum sunscreens essential for effective photoprotection. However, the long-term efficacy of sunscreen formulations is limited by the photoinstability of some organic UV filters, particularly avobenzone. Licuri oil (*Syagrus coronata*) is a naturally derived Brazilian palm oil that represents a promising alternative to medium-chain triglycerides (MCTs) as an oily core for polymeric nanocapsules. Therefore, this study aimed to develop Eudragit^®^ RS 100-based cationic nanocapsules using licuri oil for avobenzone encapsulation and to incorporate them into hyaluronic acid (HA) or xanthan gum (XG) hydrogels. The nanocapsules ranged from 125 to 158 nm, with a polydispersity index (PDI) of less than 0.2, positive zeta potential (+11 to +13 mV), and encapsulation efficiency above 98%. After 48 h of UVA exposure, the nanocapsule formulations retained 51% and 52% of their initial avobenzone content for the licuri oil- and MCT-based systems, respectively, compared with only 10% for free avobenzone, indicating a marked improvement in photostability following nanoencapsulation. Additionally, licuri oil nanocapsules exhibited increased antioxidant activity compared to MCT-based nanocapsules in both DPPH and ABTS assays. The nanocapsule suspensions were classified as non- to slightly irritating in the Hen’s Egg Test–Chorioallantoic Membrane (HET-CAM) assay. After incorporation into the hydrogels, the resulting formulations exhibited pseudoplastic and thixotropic behavior, with HA-based hydrogels providing higher UV absorption than XG-based hydrogels. Furthermore, no transdermal permeation of avobenzone was observed from either the HA- or XG-based hydrogel formulations, and the HA hydrogel containing licuri oil nanocapsules showed higher stratum corneum retention. These findings demonstrate the potential of licuri oil as an effective alternative to MCTs as the oily core of polymeric nanocapsules for topical formulations containing encapsulated avobenzone.

## 1. Introduction

Solar ultraviolet (UV) radiation exposure is a major contributor to sun-induced skin damage and photoaging, with UVB inducing direct epidermal lesions and UVA generating reactive oxygen species [1,2,3]. Broad-spectrum sunscreens remain the primary protective strategy, suppressing UV-induced oxidative stress and photoaging [1,4]. Organic UV filters, such as octocrylene, homosalate, and avobenzone, are widely incorporated into these formulations [5,6,7]. However, some UV filters are highly photounstable, such as avobenzone, which undergoes a light-induced enol-to-keto tautomeric shift that compromises its protective efficacy [8]. To address these limitations, nanoencapsulation of avobenzone is a promising formulation strategy [3,9]. Polymeric nanocarriers can physically isolate the filter from the external environment, thereby suppressing photodegradation, modulating release kinetics, and limiting systemic absorption, which ensures prolonged, localized, and safer cutaneous photoprotection [1,8,10].

Polymeric nanocapsules are particularly promising for topical delivery. These systems consist of an oily core surrounded by a polymeric shell, typically ranging from 1 to 1000 nm, which enables efficient incorporation of lipophilic compounds while protecting encapsulated molecules from external degradation and enabling targeted delivery to specific skin layers with controlled release profiles [11,12,13]. Within this group, cationic polymers such as Eudragit^®^ RS 100 are of particular interest. This polymer is composed of poly(ethyl acrylate-co-methyl methacrylate-co-trimethylammonium ethyl methacrylate chloride) at a molar ratio of 1:2:0.1, with 4.5–6.8% quaternary ammonium residues responsible for its cationic character. This permanent positive charge may facilitate interactions with negatively charged skin components, which could potentially prolong nanoparticle residence time [14,15,16].

Beyond the polymeric shell, the oily core also plays a key structural role in polymeric nanocapsules, as its composition and concentration can influence particle size, drug encapsulation, release profile, and formulation stability [17,18,19]. Therefore, selecting an appropriate oil is essential for the development of efficient nanocarriers. Among the potential oily components, oil extracted from the kernels of *Syagrus coronata* (Mart.) Becc., popularly known as “licuri”, represents an attractive alternative. This palm species is a key ecological component of the semiarid Caatinga biome in Brazil [20,21,22,23]. Its oil is typically obtained through cold mechanical pressing, a solvent-free method that preserves low initial acidity and the seed’s native bioactive compounds while minimizing environmental impact [20,22,24,25].

Licuri oil shares structural similarities with coconut oil and is characterized by a profile rich in medium-chain fatty acids, being predominantly saturated (>85%) and containing a high proportion of lauric acid (41–48%) [26,27,28]. Additionally, the therapeutic potential of the genus *Syagrus* is highlighted by the robust anti-inflammatory and analgesic activities of its fixed oil, which have been shown to significantly reduce inflammatory edema, suppress pro-inflammatory cytokines, and alleviate pain [26,29]. Licuri oil has also been evaluated as a lipophilic adjuvant in topical emulsions, showing hydrating properties comparable to those of conventional oils such as sweet almond oil, further supporting its suitability for cutaneous applications [20]. More recently, licuri oil has been explored as an oily component in nanoformulations, demonstrating anticonvulsant [30] and antifungal activities [31], as well as potential application in formulations for topical acne management [32].

Although polymeric nanocapsules offer several advantages, their low-viscosity aqueous nature limits direct skin application. Incorporation into semisolid vehicles, particularly hydrogels, is an effective strategy to provide suitable consistency, improve spreadability, and increase skin residence time [33,34,35]. In addition, the hydrogel matrix can influence the release and cutaneous distribution of encapsulated compounds, making polymer selection an important aspect of formulation design. Recent studies have shown that polymer-based hydrogels containing antioxidant nanocapsules can improve the delivery of active agents to the skin and enhance tissue protection against UVB-induced injury [1,36].

Despite the growing interest in natural oils for pharmaceutical applications, the viability of licuri oil as an alternative to medium-chain triglycerides (MCTs) for avobenzone encapsulation has not been explored. Therefore, this study aimed to develop cationic polymeric nanocapsules based on Eudragit^®^ RS 100, using licuri oil as the oily core for avobenzone encapsulation, and to incorporate these nanocarriers into different hydrogel matrices.

## 2. Results and Discussion

### 2.1. Characterization of the Polymeric Nanocapsules

**Figure 1:**
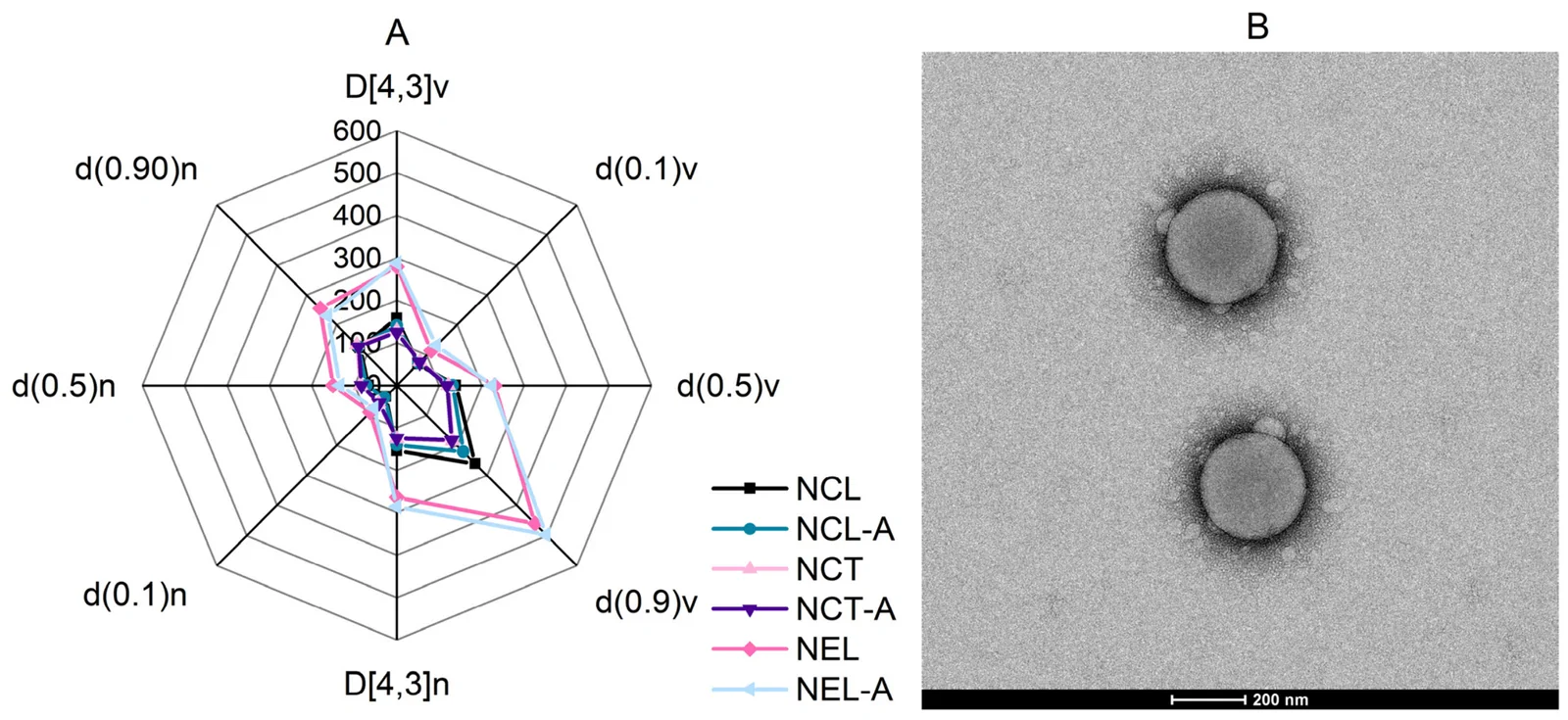
(**A**) A radar chart of the developed formulations comparing the particle size distribution parameters (D[4,3], d(0.1), d(0.5), and d(0.9)) on a volume (v) and number (n) basis, obtained via laser diffraction, and (**B**) a transmission electron micrograph of NCL-A. Scale bar = 200 nm. NCL: polymeric nanocapsule with licuri oil; NCL-A: polymeric nanocapsule with licuri oil and avobenzone; NCT: polymeric nanocapsule with medium-chain triglycerides; NCT-A: polymeric nanocapsule with medium-chain triglycerides and avobenzone; NEL: nanoemulsion with licuri oil; NEL-A: nanoemulsion with licuri oil and avobenzone.

The mean particle size of the developed formulations was recorded within the nanometer range (Table 1). The volume-weighted average diameter D[4,3] ranged from 125 nm to 158 nm for the nanocapsules and from 279 nm to 288 nm for the nanoemulsions. The diameter polydispersity (Span) remained close to 1, indicating a narrow particle size distribution across the samples. Through the dynamic light scattering technique (DLS), diameters between 131 nm and 164 nm were measured for the nanocapsules, while nanoemulsions exhibited diameters around 200 nm. The homogeneity of the particle size distribution was further confirmed by a low polydispersity index (PDI < 0.2). In addition to the volume-weighted average diameter and polydispersity data obtained by laser diffraction, the formulations may also be characterized by fingerprinting using radar charts constructed from eight variables, integrating the volume-weighted mean diameter (D[4,3]) and the cumulative diameters corresponding to 10% (d(0.1)), 50% (d(0.5)), and 90% (d(0.9)) of the particle population, expressed both on a volume (v) and number (n) basis [17]. This multidimensional fingerprinting approach was originally proposed by Bianchin et al. [17], who applied it to vegetable oil core nanocapsules to verify unimodal size distributions and rapidly exclude the presence of microscopic contamination in preformulation assays. Nanoparticulate formulations with a narrow, unimodal size distribution (low polydispersity) generate radar charts that are symmetric and nearly superimposable, with all diameter parameters, including the volume-based axes, situated within the nanometric range (below 500 nm). The volume-based axes are particularly sensitive to the presence of larger, micrometric particles; consequently, any contamination by microparticles, stable aggregates, or precipitated drug crystals would cause a pronounced distortion of the radar chart [17]. In our study, all formulations exhibited a symmetric and superimposable profile, with all volume percentiles remaining strictly below 500 nm. This symmetry and the lack of outliers on the volume axes mathematically confirm a narrow, unimodal size distribution and the absence of microscopic contamination or microaggregates. Among the formulations, the graphs (Figure 1A) reveal a similar overall pattern, with the nanoemulsions showing consistently larger particle size than the nanocapsules. Notably, replacing MCTs with licuri oil did not alter the diameter profile of the nanocapsules, indicating that the change in oily core did not compromise the size distribution of the particles. Based on nanoparticle tracking analysis (NTA) measurements from NCL-A and NCT-A, the mean diameter values were 185 ± 2 and 171 ± 3, respectively. The complementary use of laser diffraction, DLS, and NTA provides comprehensive validation in nanotechnology, as each technique relies on a distinct measurement principle. Laser diffraction is based on Mie theory, inferring particle size from the angle of scattered light, whereas DLS and NTA are based on the Stokes–Einstein equation and measure the hydrodynamic diameter of particles undergoing Brownian motion. While DLS measures the hydrodynamic diameter of particle populations, NTA tracks the Brownian motion of individual particles, allowing for better resolution of nanometric subpopulations [11,18,37]. Given these differences in measurement principles, some numerical divergence between techniques is expected; nonetheless, all values remained within the same order of magnitude and below 200 nm, confirming the nanometric size range regardless of the technique used.

The zeta potential of the nanocapsule suspensions was approximately +11 mV, which is attributed to the cationic nature of Eudragit^®^ RS 100 resulting from the presence of quaternary ammonium groups [15]. In contrast, the nanoemulsions exhibited negative zeta potential values, consistent with the absence of Eudragit^®^ RS 100 from their composition. According to previous studies, positive zeta potential values may be particularly relevant to the development of topical formulations, as electrostatic interactions between positively charged nanoparticles and the negatively charged skin components may favor their association with the skin surface [14]. This electrostatic attraction may also contribute to the formation of a localized depot or an occlusive effect on the stratum corneum, potentially prolonging the formulation’s residence time at the application site [1,34,38].

The pH values ranged from 3.4 to 4.8. Notably, compared with those containing MCTs, the pH values of nanocapsules containing licuri oil were significantly greater. The licuri oil nanoemulsion exhibited a pH similar to that of the MCT-based formulations, suggesting that the higher pH observed for the licuri oil nanocapsules may be associated with the combined presence of licuri oil and Eudragit^®^ RS 100 in the nanocapsule formulation, rather than with the intrinsic properties of licuri oil alone.

With respect to avobenzone content and encapsulation efficiency, avobenzone content reached 93.39 ± 0.60% for NCL-A and 92.77 ± 0.64% for NCT-A. Furthermore, the encapsulation efficiency exceeded 98% for both systems (98.77 ± 0.38% and 98.90 ± 0.46%, respectively). Such high entrapment efficiency (>98%) is consistent with that observed for lipophilic compounds encapsulated in polymeric nanocapsules prepared via the interfacial deposition method [34,38]. These findings indicate a strong affinity between the active ingredient and the polymeric matrix, demonstrating the potential of licuri oil to act as an oily core in the development of polymeric nanocapsules.

Evaluation of the NCL-A formulation by transmission electron microscopy revealed regular spherical structures (Figure 1B) with an average particle diameter of approximately 200 nm, in agreement with the results obtained by laser diffraction and DLS. Similar spherical morphology and a close correlation between microscopic observations and light scattering diameters have been documented for other vegetable oil core cationic nanocapsules prepared with Eudragit^®^ RS100 [39].

### 2.2. Photostability Evaluations

Photostability is a vital parameter in sunscreen design, as the photodegradation of active filters not only results in a loss of UV protection but can also lead to the generation of phototoxic and photoallergic byproducts on the skin surface [8,40]. The photostability of avobenzone is influenced by keto-enol tautomerism, with UV exposure shifting the balance toward the keto form, which is absorbed in the UVC range, thereby reducing its effectiveness [41,42]. Given that previous studies have demonstrated the advantages of nanoencapsulation in enhancing sunscreen efficacy [8,9], we evaluated the photostability of avobenzone encapsulated with licuri oil.

**Figure 2:**
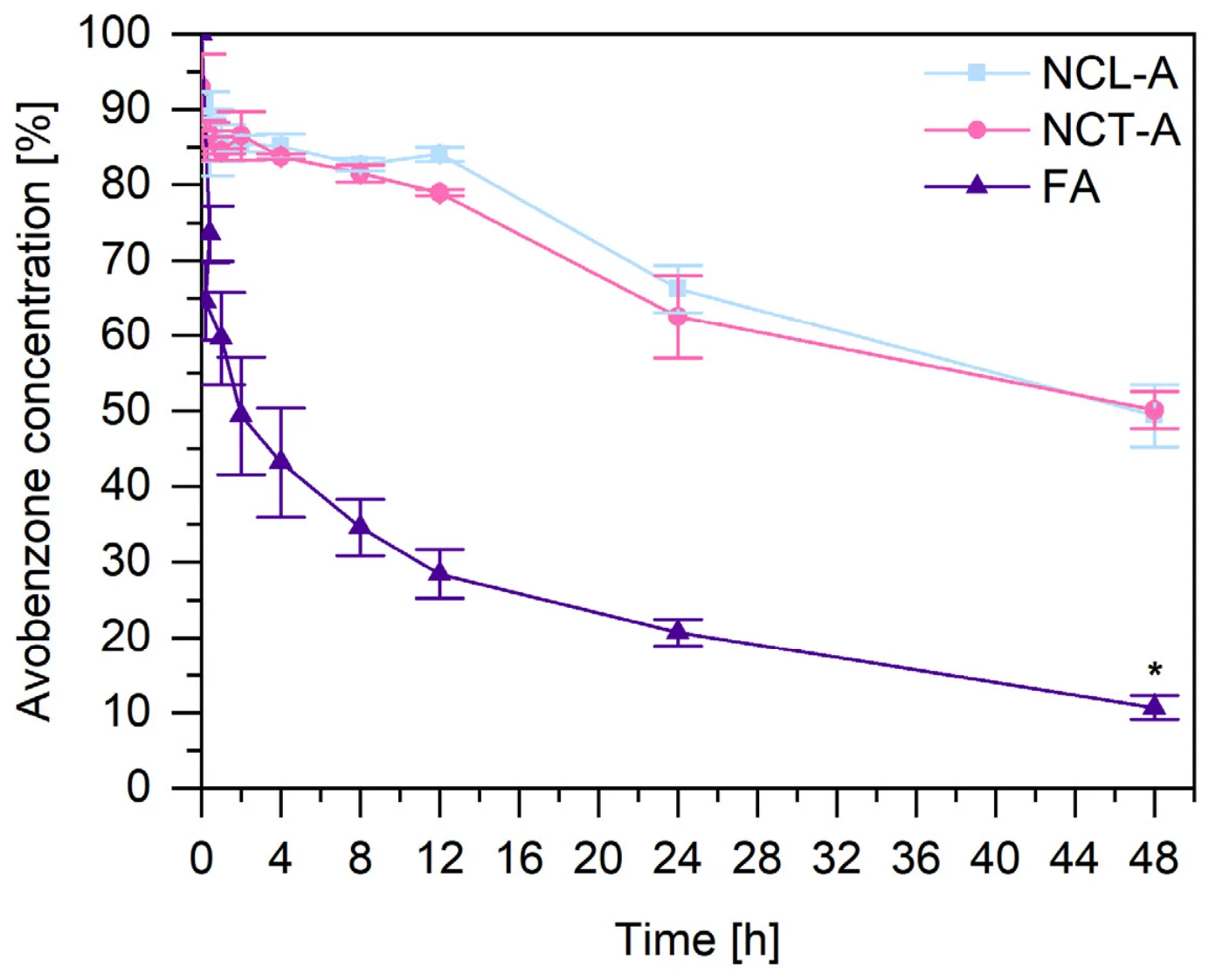
Avobenzone concentration (%) over time in the developed formulations (NCL-A, NCT-A, and free avobenzone). The values are expressed as the mean ± standard deviation (*n* = 3). Statistically significant differences were determined by one-way ANOVA followed by Tukey’s post hoc test (*p* ≤ 0.05). * Indicates a significant difference compared to free avobenzone (*p* ≤ 0.05). No statistically significant difference was observed between NCL-A and NCT-A (*p* > 0.05). NCL-A: polymeric nanocapsule with licuri oil and avobenzone; NCT-A: polymeric nanocapsule with medium-chain triglycerides and avobenzone.

The concentration of avobenzone in its free form (FA, acetonitrile/water, 1:1, *v*/*v*) decreased from 100% to 10% after 48 h of UVA exposure (Figure 2). In contrast, the photostability of nanoencapsulated avobenzone improved, with concentrations decreasing from 93% to 51% when encapsulated with licuri oil and from 97% to 52% when encapsulated with MCTs over the same period. The statistically significant difference in avobenzone content after 48 h of UVA exposure (*p* ≤ 0.05) was confirmed between the free avobenzone group and both nanocapsule formulations, confirming that encapsulation enhances avobenzone photostability. In contrast, no significant difference (*p* > 0.05) was found between NCL-A and NCT-A (51% and 52% remaining avobenzone, respectively), indicating that, under the conditions evaluated, the stabilizing effect was not significantly influenced by the oily core used.

Based on reported solar UVA irradiance reaching the Earth’s surface at midday under clear-sky conditions (20–60 W m^−2^, from winter to summer [43]), corresponding to a cumulative UVA dose of 7.2–21.6 J cm^−2^ over 1 h of exposure, the cumulative dose applied in the present study (38.02 J cm^−2^) would be reached after approximately 1.8–5.3 h of continuous midday solar exposure, depending on the solar UVA irradiance. Considering that sunscreens are recommended to be reapplied every 2 h of sun exposure to maintain adequate protection [44], this comparison provides a relevant radiometric context for the UVA dose used in the present photostability study.

These results are consistent with those reported by Cozzi et al. [9], who reported a 28% decrease in UVA protection and a 27% decrease in UVB protection for the free forms of avobenzone and octocrylene after 4 h of UV exposure. In contrast, the protection of the nanoencapsulated form was not significant. The improved stability of nanoencapsulated avobenzone is mainly attributed to the shielding effect and physical barrier provided by the polymer matrix that encloses the UV filter [45].

### 2.3. In Vitro Antioxidant Assays

**Figure 3:**
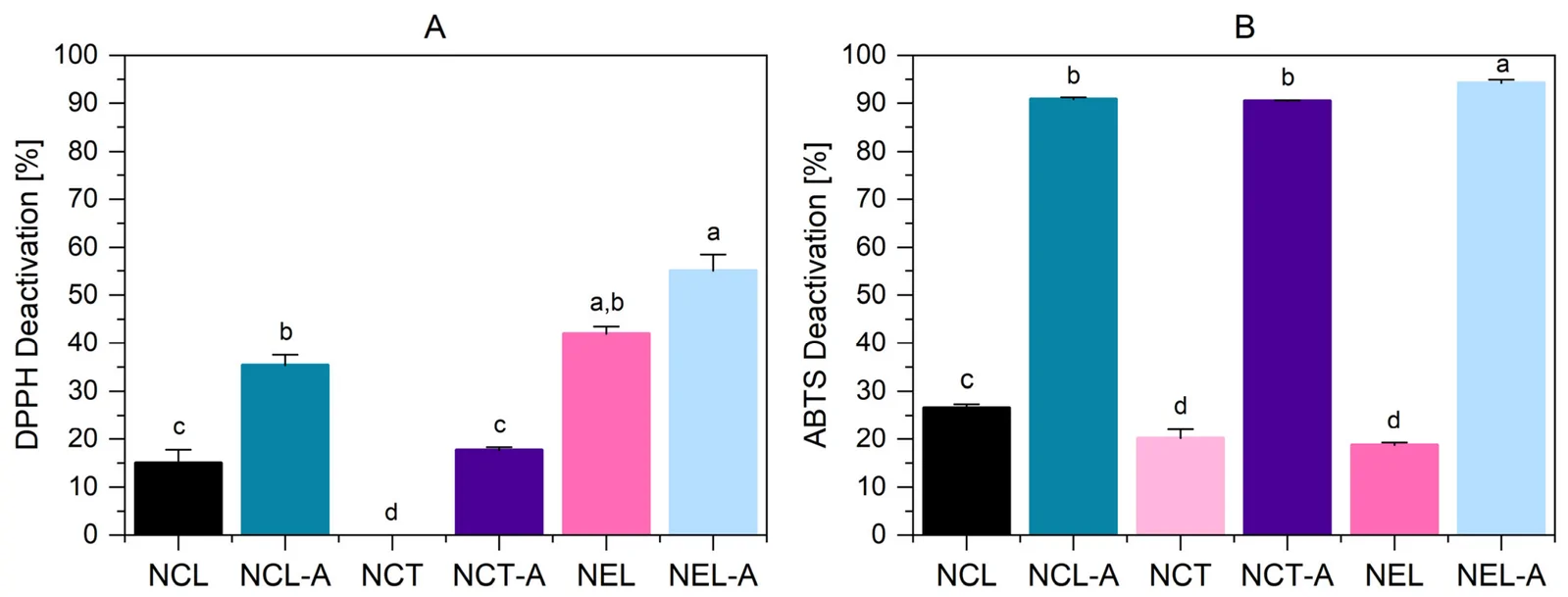
The percentages (%) of (**A**) DPPH radical deactivation and (**B**) ABTS radical deactivation of the developed formulations. The values are expressed as the mean ± standard error (*n* = 3). Different letters indicate statistically significant differences among groups (*p* ≤ 0.05; one-way ANOVA followed by Tukey’s post hoc test). NCL: polymeric nanocapsule with licuri oil; NCL-A: polymeric nanocapsule with licuri oil and avobenzone; NCT: polymeric nanocapsule with medium-chain triglycerides; NCT-A: polymeric nanocapsule with medium-chain triglycerides and avobenzone; NEL: nanoemulsion with licuri oil; NEL-A: nanoemulsion with licuri oil and avobenzone.

The incorporation of avobenzone enhanced the antioxidant activity in most formulations, although the effect varied by assay and matrix type. In the DPPH assay (Figure 3A), NEL-A showed the highest scavenging activity (55.00%), followed by NEL (42.00%). These did not differ significantly (*p* ≤ 0.05), indicating that avobenzone did not contribute to this matrix. This finding points to licuri oil itself as the primary source of hydrogen-donating activity, consistent with its reported phenolic content and DPPH capacity [23]. The nanoemulsion outperformed the nanocapsule for the same oil, both with (NEL-A, 55.00% vs. NCL-A, 35.35%) and without avobenzone (NEL, 42.00% vs. NCL, 14.98%), indicating greater accessibility of the free oil phase than of the oil entrapped within the polymeric nanocapsule matrix. NCT showed no DPPH activity (0.00%), whereas the activity of NCT-A reached 17.68%. The residual DPPH activity observed in NCT-A is therefore likely attributable to avobenzone rather than to MCT.

The results of the ABTS assay (Figure 3B) revealed a stronger and more consistent effect of avobenzone. All three pairs increased significantly: NCL (26.58%) to NCL-A (90.85%), NCT (20.19%) to NCT-A (90.45%), and NEL (18.76%) to NEL-A (94.25%) (*p* ≤ 0.05). This increase across independent oil matrices indicates that avobenzone is the primary driver of ABTS activity, though the mechanism remains incompletely understood.

Interestingly, NEL exhibited high DPPH scavenging activity (42.00%) but a lower ABTS value (18.76%), suggesting that the bioactive compounds in licuri oil may be more effective in specific free-radical scavenging mechanisms or under particular reaction conditions. This divergent behavior between the assays highlights the influence of the antioxidants’ chemical composition and properties, as well as the formulation’s complex reaction environment.

### 2.4. In Vitro Study of Irritating Potential (HET-CAM)

Preclinical toxicological screening is a fundamental step in the development of new topical formulations to ensure biocompatibility prior to human exposure, particularly for products intended for daily facial use, where contact with sensitive cutaneous tissue and ocular mucosa is highly likely. Therefore, the HET-CAM test was used as an alternative in vitro model to screen the irritation potential of the formulations [13,46,47]. The positive controls, 0.1 mol L^−1^ NaOH and 1% (*w*/*v*) sodium lauryl sulfate, were classified as an extreme irritant (IS: 13.44 ± 0.20) and a moderate irritant (IS: 7.85 ± 2.44), respectively, based on the presence of hemorrhage, coagulation, and vasoconstriction. In contrast, the negative control (0.9% *w*/*v* NaCl) did not exhibit any of the evaluated vascular effects (IS: 0.00 ± 0.00).

**Figure 4:**
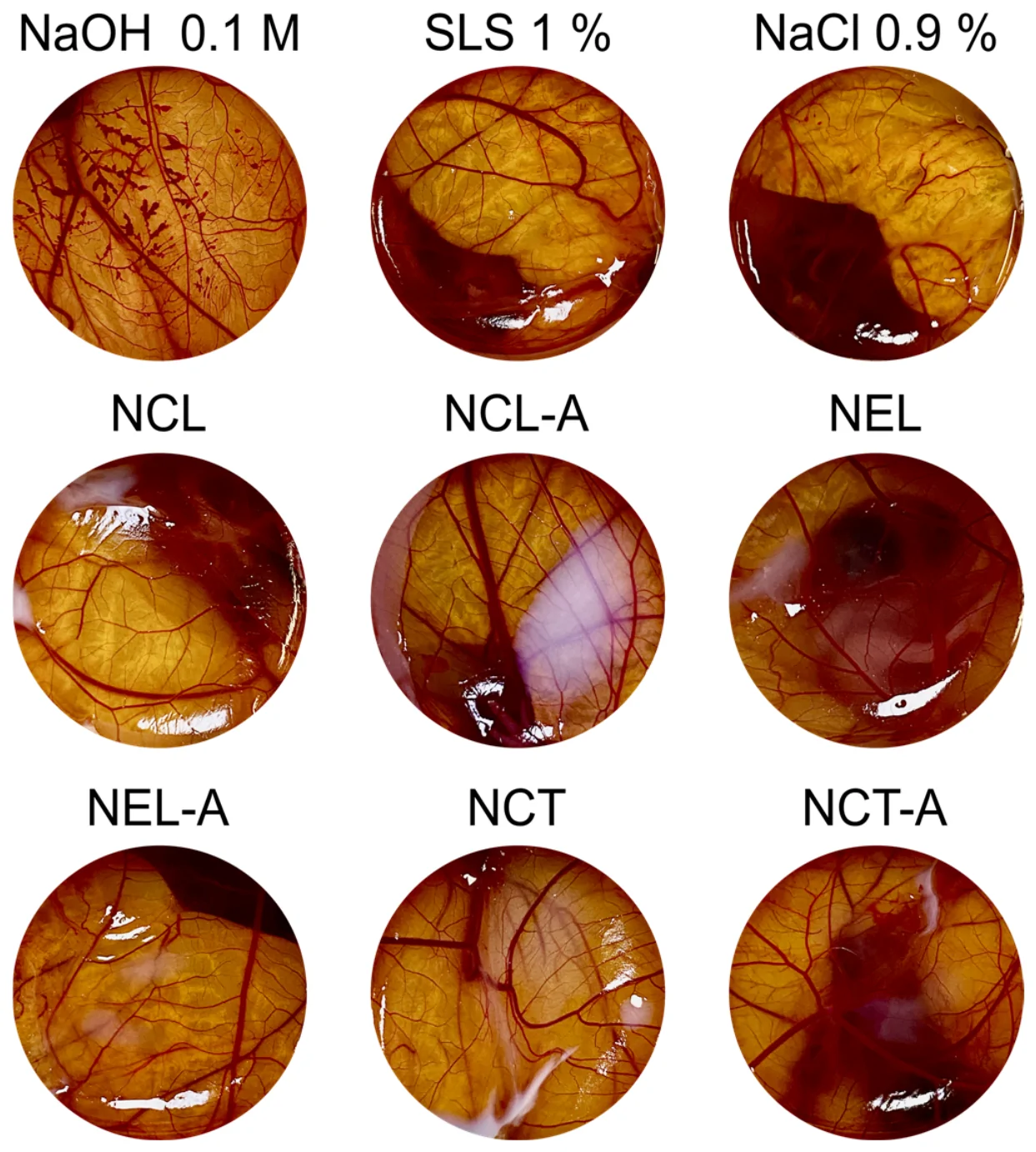
Representative images of the chorioallantoic membrane (CAM) after 5 min of exposure to the positive control (0.1 mol L^−1^ NaOH and 1% SLS), the negative control (0.9% NaCl), and the samples (NCL, NCL-A, NEL, NEL-A, NCT, and NCT-A). NCL: polymeric nanocapsule with licuri oil; NCL-A: polymeric nanocapsule with licuri oil and avobenzone; NCT: polymeric nanocapsule with medium-chain triglycerides; NCT-A: polymeric nanocapsule with medium-chain triglycerides and avobenzone; NEL: nanoemulsion with licuri oil; NEL-A: nanoemulsion with licuri oil and avobenzone.

Among the samples analyzed, NCL and NEL-A were classified as slight irritants (IS: 2.90 ± 0.61 and 1.10 ± 0.26, respectively), as illustrated in Figure 4. The remaining formulations were classified as nonirritating, with IS values of 0.90 ± 0.77 for NCL-A, 0.60 ± 0.82 for NEL, 0.77 ± 0.47 for NCT, and 0.90 ± 0.69 for NCT-A. Overall, none of the formulations reached the extreme irritant category, supporting their safety for topical application. These findings are consistent with previous safety assessments of licuri oil, which reported no significant cytotoxic or genotoxic effects, further supporting its suitability as a biocompatible component for topical nanotechnology-based formulations [48].

### 2.5. Physicochemical Characterization of the Hydrogels

Hydrogels containing licuri oil nanocapsule suspensions or nanoemulsions, with or without avobenzone, were characterized for macroscopic appearance, mean nanoparticle diameter after incorporation, polydispersity index, pH, and avobenzone content. Rheological behavior was additionally evaluated for hydrogels containing nanocapsule suspensions. Physicochemical characterization at the hydrogel stage focused on the licuri oil formulation, given that suspension-level comparisons already established comparable physicochemical behavior between licuri oil and MCT nanocapsules.

Hydrogels prepared with hyaluronic acid or xanthan gum in water were initially transparent and homogeneous. After incorporating nanocapsule suspensions containing licuri oil or MCT, as well as licuri oil nanoemulsions with or without avobenzone, all formulations exhibited a white coloration. Hyaluronic acid-based formulations containing nanostructured systems maintained a glossy and homogeneous appearance. In contrast, the xanthan gum-based formulations exhibited an opaque and heterogeneous appearance, even after homogenization during preparation.

**Figure 5:**
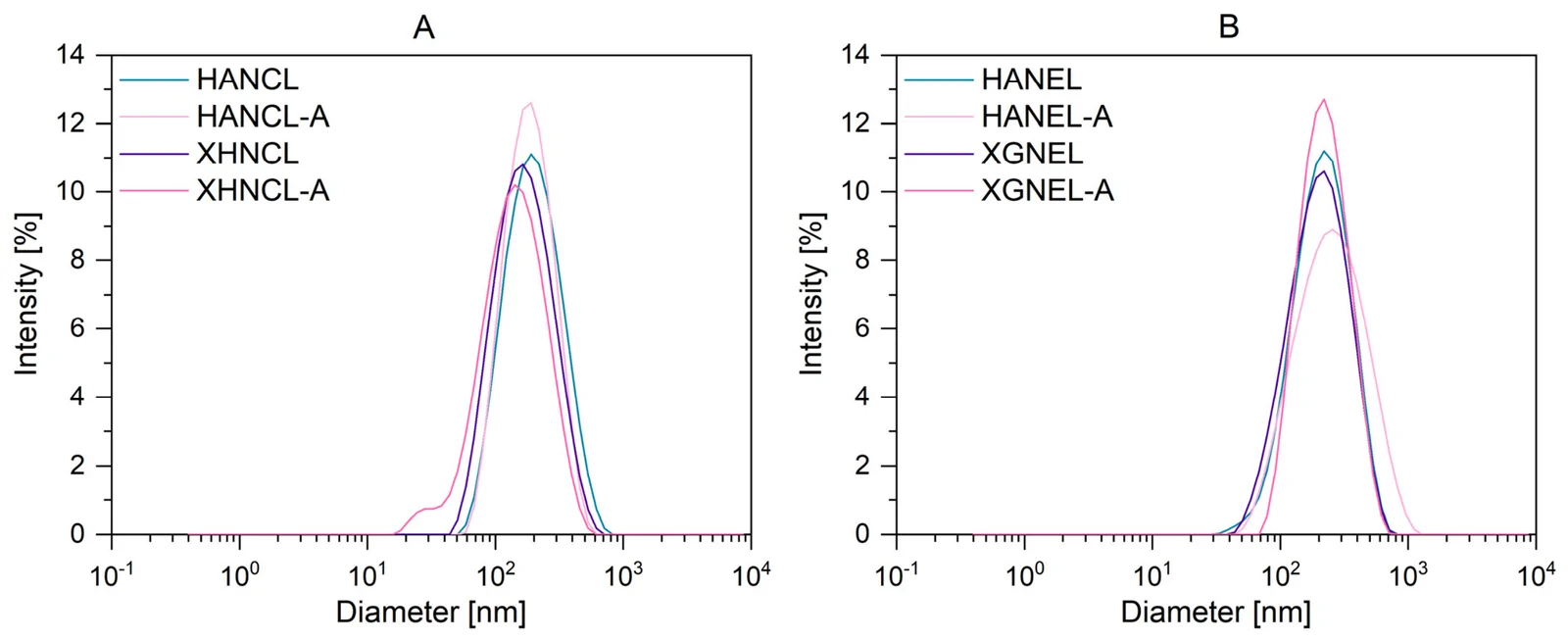
Particle size distribution based on the intensity of (**A**) nanocapsules and (**B**) nanoemulsions incorporated into hyaluronic acid (HA) and xanthan gum (XG) hydrogels. HANCL: hyaluronic acid hydrogel containing polymeric nanocapsules with licuri oil; HANCL-A: hyaluronic acid hydrogel containing polymeric nanocapsules with licuri oil and avobenzone; XGNCL: xanthan gum hydrogel containing polymeric nanocapsules with licuri oil; XGNCL-A: xanthan gum hydrogel containing polymeric nanocapsules with licuri oil and avobenzone; HANEL: hyaluronic acid hydrogel containing nanoemulsion with licuri oil; HANEL-A: hyaluronic acid hydrogel containing nanoemulsion with licuri oil and avobenzone; XGNEL: xanthan gum hydrogel containing nanoemulsion with licuri oil; XGNEL-A: xanthan gum hydrogel containing nanoemulsion with licuri oil and avobenzone.

The mean diameter, polydispersity index, pH, and avobenzone content were evaluated after the nanocapsule suspension and nanoemulsion were incorporated into the hydrogels. The *z*-average analysis revealed particle sizes ranging from approximately 117 to 204 nm (Table 2), indicating that the developed systems are on a nanometric scale. Variations in size were observed depending on the polymeric matrix (HA or XG) and the incorporation of avobenzone; however, all formulations maintained a consistent nanometric profile. With respect to the polydispersity index, all formulations exhibited values below 0.3, indicating a narrow particle size distribution and high structural homogeneity of the nanoparticles after dispersion in the hydrogel (Figure 5).

The formulations exhibited pH values ranging from approximately 5.5 to 6.2. The HANCL and HANEL formulations had slightly lower pH values (approximately 5.5–5.6), whereas the other formulations maintained values in a slightly higher range, between 5.9 and 6.2 (Table 2). Compared with the corresponding nanoformulations (Table 1), the hydrogel formulations exhibited higher pH values. This difference may be related to changes in the ionic environment and acid–base equilibrium of the aqueous phase following incorporation of the nanoformulations into the HA and XG matrices. The ionizable groups present in these polysaccharides may contribute to the observed pH shift, as their protonation state can be influenced by the composition of the surrounding medium [49,50,51]. Since similar behavior was observed in both nanocapsule- and nanoemulsion-containing hydrogels, this effect may be associated with the incorporation of the nanocapsule or nanoemulsion formulation into the polysaccharide matrix rather than with a specific component of the nanocapsules [3,11,52]. Overall, the formulations exhibited pH values of approximately 6.0, which are considered appropriate for topical application, ensuring compatibility with the skin’s physiological pH and minimizing the risk of irritation [53].

The avobenzone content of the hyaluronic acid-based formulations remained close to 100% (Table 2), whereas that of the xanthan gum-based formulations was 87.33 ± 2.38% for the nanoemulsions and 94.11 ± 0.99% for the nanocapsule suspensions. The deviation observed across the avobenzone content between the hyaluronic acid- and xanthan gum-based formulations is likely attributed to matrix-specific differences in extraction efficiency related to the structural conformation of the two polysaccharides. Xanthan gum adopts a rigid tertiary conformation due to its ordered double-helical structure, forming a more organized network, whereas hyaluronic acid possesses a flexible tertiary conformation due to its random-coil structure [49]. As a result, xanthan gum is known to interact with and retain hydrophobic compounds, which can reduce their quantitative recovery during HPLC analysis [54]. Given the lipophilic nature of avobenzone and the rigid structure of xanthan gum, similar retention within the polymer network may partially explain the lower avobenzone content values observed in xanthan gum-based hydrogels, whereas the more flexible, less structured hyaluronic acid matrix likely enabled more efficient and reproducible avobenzone extraction.

### 2.6. Rheological Analysis of the Hydrogels

**Figure 6:**
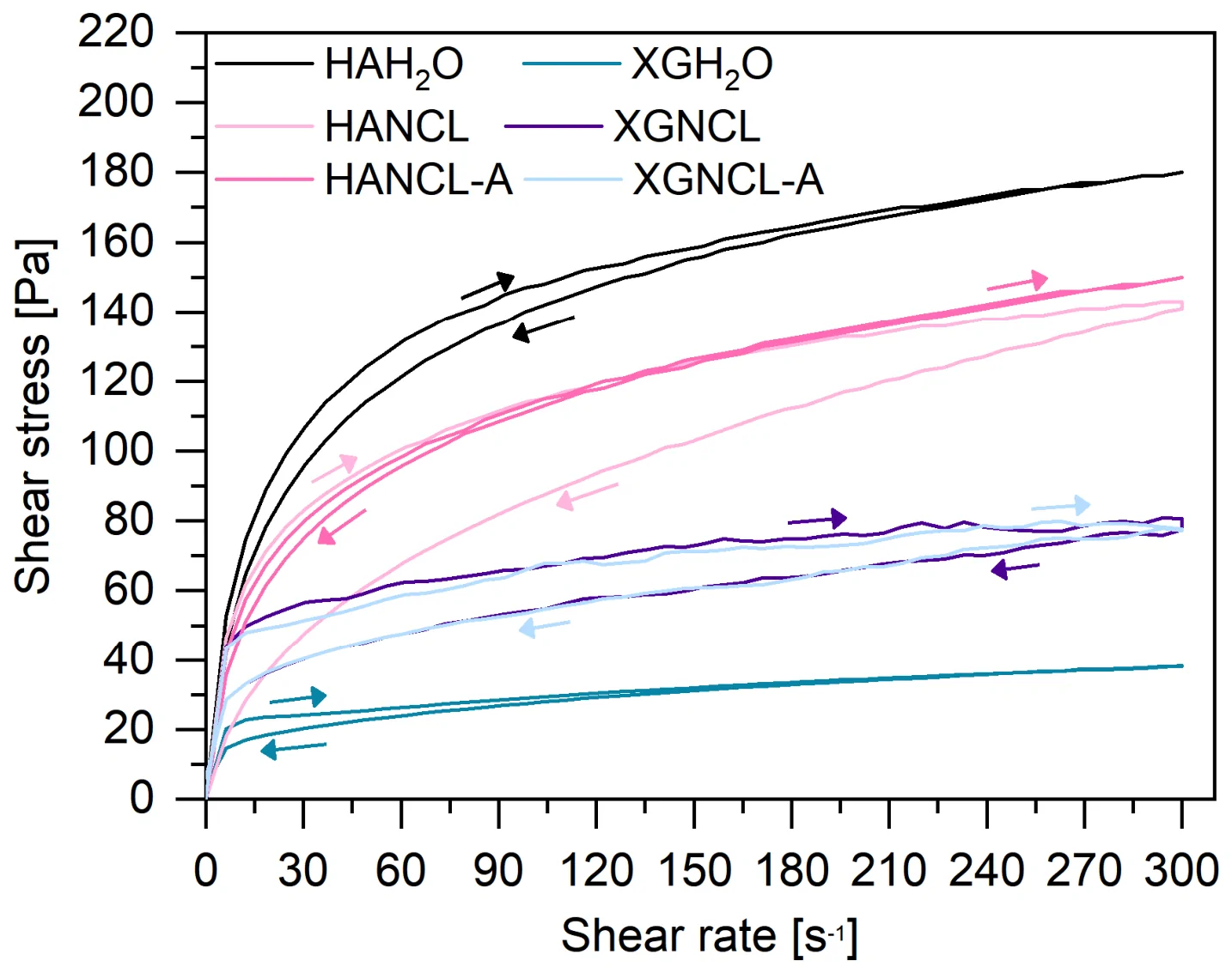
Flow curves of the hydrogels (*n* = 3). The arrows indicate the outward and return sweeps, for each formulation. HAH_2_O: hyaluronic acid hydrogel with water; HANCL: hyaluronic acid hydrogel containing polymeric nanocapsules with licuri oil; HANCL-A: hyaluronic acid hydrogel containing polymeric nanocapsules with licuri oil and avobenzone; XGH_2_O: xanthan gum hydrogel with water; XGNCL: xanthan gum hydrogel containing polymeric nanocapsules with licuri oil; XGNCL-A: xanthan gum hydrogel containing polymeric nanocapsules with licuri oil and avobenzone.

The flow curves (Figure 6) demonstrated that all the developed formulations exhibit non-Newtonian, pseudoplastic behavior, characterized by a reduction in viscosity with increasing shear rate and a nonlinear relationship between shear stress and shear rate [55]. This rheological profile is desirable for topical photoprotective formulations, as it promotes the formation of stable, uniform films on the skin surface [56]. This pseudoplastic flow, widely reported in other topical hydrogels containing nanostructured systems, prevents formulation runoff and ensures uniform distribution of the active substance upon cutaneous application [52].

Formulations containing xanthan gum presented lower shear stress values than those prepared with hyaluronic acid, indicating lower consistency. This behavior may be associated with the more heterogeneous appearance observed for the xanthan gum-based hydrogels in the present study, which could reflect differences in the organization of the polymeric network.

All formulations exhibited thixotropic behavior, characterized by a time-dependent reduction in viscosity under shear stress and a gradual structural recovery after stress removal. According to previous studies, thixotropic behavior may facilitate formulation spreadability and contribute to its retention on the skin [11,57]. In the context of sun care technology, the literature also suggests that thixotropic behavior may help balance spreadability and bioadhesion, thereby favoring the localization of the formulation on the stratum corneum [36].

Hysteresis area values obtained from the rheological curves indicated that formulations containing nanocapsule suspensions exhibited higher thixotropy than the corresponding polymeric hydrogels without nanoparticles. In general, formulations with larger hysteresis areas demonstrated improved spreadability, whereas the xanthan gum hydrogels without nanoparticles (XGH_2_O), which exhibited the lowest hysteresis area, may present reduced adherence to the skin [56]. These results suggest that incorporating nanocapsules enhances the hydrogels’ thixotropic properties, yielding more suitable rheological characteristics for topical application.

### 2.7. Evaluation of the UV Radiation Absorption Capacity of the Hydrogels

**Figure 7:**
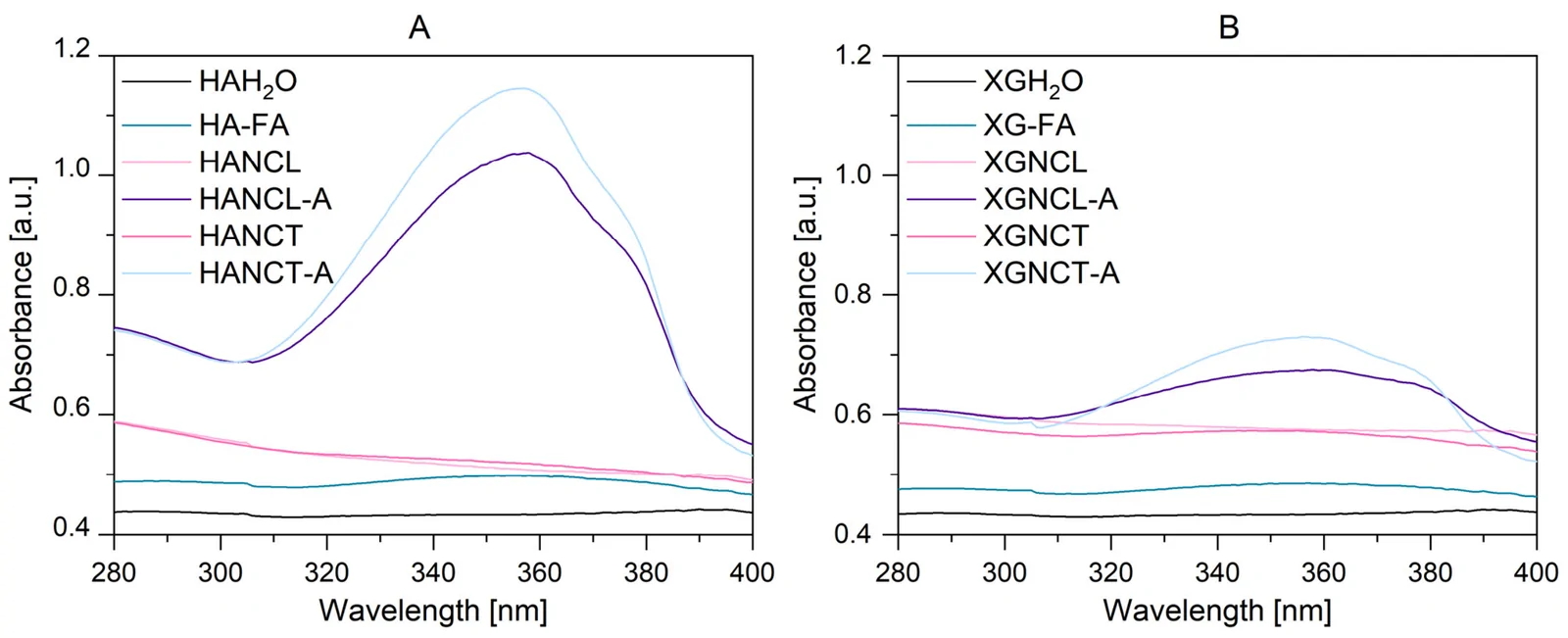
UV absorbance spectra (280–400 nm) of hydrogels based on (**A**) hyaluronic acid and (**B**) xanthan gum (*n* = 6). HAH_2_O: hyaluronic acid hydrogel with water; HA-FA: hyaluronic acid hydrogel with free avobenzone; HANCL: hyaluronic acid hydrogel containing polymeric nanocapsules with licuri oil; HANCL-A: hyaluronic acid hydrogel containing polymeric nanocapsules with licuri oil and avobenzone; HANCT: hyaluronic acid hydrogel containing polymeric nanocapsules with medium-chain triglycerides; HANCT-A: hyaluronic acid hydrogel containing polymeric nanocapsules with medium-chain triglycerides and avobenzone; XGH_2_O: xanthan gum hydrogel with water; XG-FA: xanthan gum hydrogel with free avobenzone; XGNCL: xanthan gum hydrogel containing polymeric nanocapsules with licuri oil; XGNCL-A: xanthan gum hydrogel containing polymeric nanocapsules with licuri oil and avobenzone; XGNCT: xanthan gum hydrogel containing polymeric nanocapsules with medium-chain triglycerides; XGNCT-A: xanthan gum hydrogel containing polymeric nanocapsules with medium-chain triglycerides and avobenzone.

The UV absorption spectra of the formulations in the 280–400 nm range are shown in Figure 7. The absorption profiles of the hyaluronic acid-based hydrogels are shown in Figure 7A. Formulations containing encapsulated avobenzone (HANCL-A and HANCT-A) exhibited the highest absorption intensities, with maxima at approximately 357–358 nm, corresponding to the characteristic absorption region of avobenzone [42]. In contrast, formulations without avobenzone but containing nanocapsules exhibited increased absorbance across the entire wavelength range evaluated compared with the corresponding hydrogels without nanocapsules. As previously described in the literature, this apparent absorption is likely a secondary effect arising from light scattering by the colloidal particles [3,34]. Compared with the encapsulated systems, the free avobenzone hydrogel (HA-FA) also exhibited an absorption maximum near 359 nm, although with a lower intensity. The absorption intensity of the control formulation (HAH_2_O) was low throughout the evaluated range. Similar behavior was observed for the xanthan gum-based hydrogels (Figure 7B). Encapsulated avobenzone formulations (XGNCL-A and XGNCT-A) exhibited absorption maxima within the characteristic avobenzone range. As observed for the hyaluronic acid-based hydrogels, blank nanocapsule-containing formulations without sunscreen showed a similar scattering-related absorbance increase across the spectrum. Compared with the encapsulated formulations, the free avobenzone hydrogel (XG-FA) also presented a lower absorption intensity.

Overall, compared with xanthan gum-based formulations, hyaluronic acid-based hydrogels exhibited higher UV absorption intensity. This behavior may be attributed to the greater film-forming properties of hyaluronic acid, which likely facilitate the formation of more uniform films and improve the distribution of the nanostructures. In contrast, xanthan gum forms viscous and turbid gels and has been reported to exhibit poor film-forming ability, which may contribute to the lower UV absorption observed [58].

### 2.8. Evaluation of In Vitro Skin Penetration and Permeation of Avobenzone from the Hydrogels

**Figure 8:**
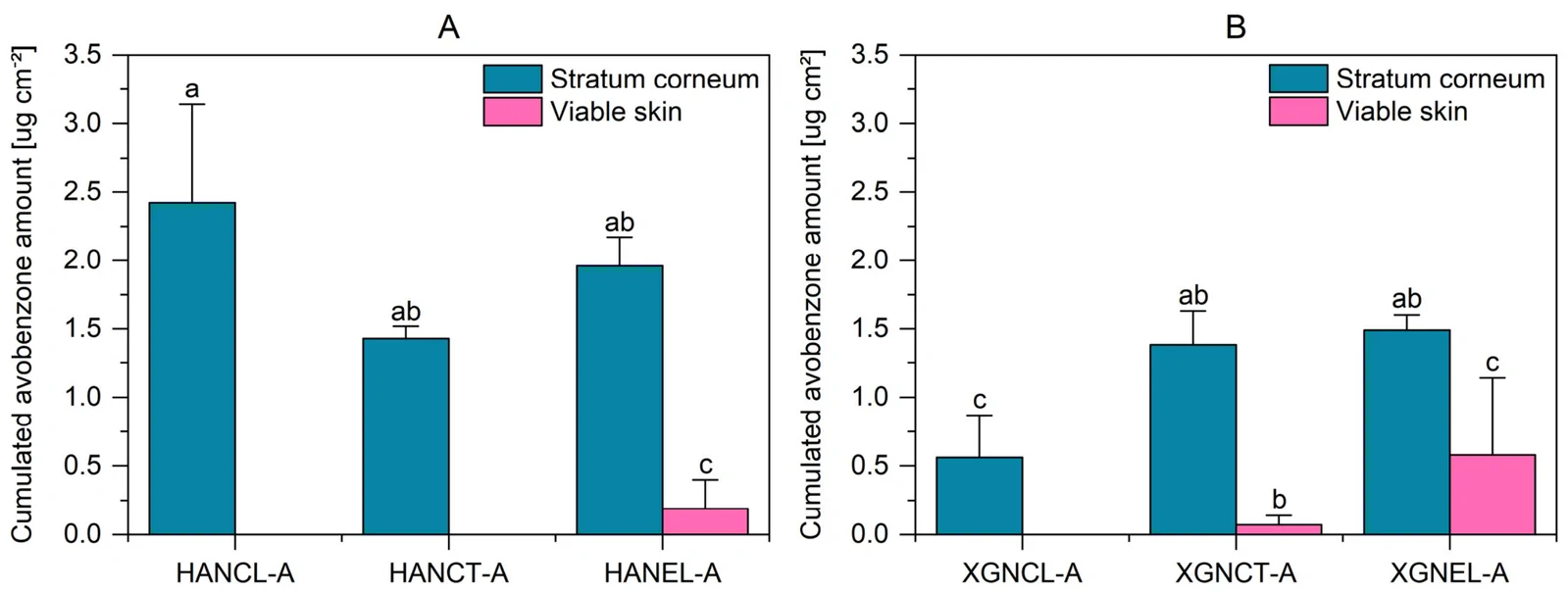
The total amount of avobenzone retained in the stratum corneum and viable skin from hyaluronic acid-based (**A**) and xanthan gum-based (**B**) hydrogels (*n* = 4). Different letters indicate statistically significant differences among groups (*p* ≤ 0.05), as determined by one-way ANOVA followed by Tukey’s test. HANCL-A: hyaluronic acid hydrogel containing polymeric nanocapsules with licuri oil and avobenzone; HANCT-A: hyaluronic acid hydrogel containing polymeric nanocapsules with medium-chain triglycerides and avobenzone; HANEL-A: hyaluronic acid hydrogel containing nanoemulsion with licuri oil and avobenzone; XGNCL-A: xanthan gum hydrogel containing polymeric nanocapsules with licuri oil and avobenzone; XGNCT-A: xanthan gum hydrogel containing polymeric nanocapsules with medium-chain triglycerides and avobenzone; XGNEL-A: xanthan gum hydrogel containing nanoemulsion with licuri oil and avobenzone.

Photoprotective formulations are widely used to minimize the harmful effects of excessive UV radiation, and their efficacy depends on the formation of a uniform, stable, and adherent film on the skin surface that absorbs, scatters, and reflects incident UV radiation [9,34,56]. However, organic UV filters such as avobenzone may present chemical instability and penetrate into deeper skin layers [9]. Therefore, in this study, the influence of nanostructured formulations on the penetration and permeation of avobenzone into the skin was evaluated. Hydrogels containing nanocapsule suspensions with licuri oil (HANCL-A, XGNCL-A) or medium-chain triglycerides (HANCT-A, XGNCT-A), as well as licuri oil nanoemulsions used as controls (HANEL-A and XGNEL-A), were evaluated for avobenzone retention in the skin layers and permeation through the skin. Avobenzone quantification in the receptor medium was performed at 30 min and 1, 2, 4, 6, and 8 h. Throughout the 8 h in vitro experiment, no avobenzone was detected in the receptor compartment for any formulation, indicating no skin permeation. This lack of percutaneous absorption is a crucial safety outcome for sun care products, as active UV filters must remain localized on the skin surface to exert their photoprotective function without reaching the systemic circulation [9,59]. Figure 8 shows the amount of avobenzone retained in the stratum corneum and viable skin after the application of hyaluronic acid-based (Figure 8A) and xanthan gum-based hydrogels (Figure 8B).

With respect to the hyaluronic acid formulations, avobenzone was detected in viable skin only for the nanoemulsion formulation (HANEL-A). In contrast, formulations containing nanocapsules (HANCL-A and HANCT-A) showed no penetration into this layer. No significant differences were observed in stratum corneum retention among the formulations, indicating similar deposition profiles for nanocapsules prepared with licuri oil or MCT. With respect to the xanthan gum formulations, avobenzone was detected in viable skin after the application of the nanoemulsion (XGNEL-A) and MCT nanocapsules (XGNCT-A) but not after the application of the licuri oil nanocapsules (XGNCL-A). Similarly, no significant differences were observed in stratum corneum retention among the nanocapsule formulations.

The greater penetration observed for nanoemulsion formulations may be associated with structural differences between nanoemulsions and polymeric nanocapsules, as nanocapsules tend to exhibit greater affinity for the stratum corneum and promote greater retention on the skin surface [33,60]. Similar findings were reported by Puglia et al. [59], who reported lower skin permeation of nanoencapsulated avobenzone than of nanoemulsions. In addition, Cozzi et al. [9] demonstrated that nanoencapsulation reduced the penetration of avobenzone into deeper skin layers, promoting its retention on the skin surface.

Among the evaluated formulations, HANCL-A promoted greater avobenzone accumulation in the stratum corneum than XGNCL-A did, indicating that the hydrogel matrix influenced the cutaneous deposition of the encapsulated UV filter. The higher consistency of the hyaluronic acid hydrogels may have favored longer retention of the formulation on the skin surface, thereby contributing to greater avobenzone retention within the stratum corneum. Overall, the absence of avobenzone in the receptor medium over the 8 h in vitro study period underscores the ability of polymeric nanostructured systems to retain the UV filter within the superficial skin layers, thereby contributing to safer topical photoprotective formulations.

## 3. Materials and Methods

### 3.1. Materials

Licuri oil (*Syagrus coronata*—SisGen n° A08E18B) was kindly donated by the Cooperativa de Produção da Região de Piemonte da Diamantina (COOPES) (Capim Grosso, Brazil). The oil was extracted mechanically using a hydraulic press. Avobenzone was obtained from JoviiCosmecêutica (Cachoeirinha, Brazil). High-molecular-weight hyaluronic acid and xanthan gum were obtained from MCassab (São Paulo, Brazil) and Sarfam (São Paulo, Brazil), respectively. Capric/caprylic triglycerides were purchased from Delaware (Porto Alegre, Brazil). Eudragit^®^ RS 100 was obtained from Labsynth (São Paulo, Brazil). Polysorbate 80 (Tween 80) was obtained from Synth (Diadema, Brazil). Imidazolidinyl urea was obtained from Fitonfarma (Porto Alegre, Brazil). Acetonitrile was of HPLC grade and was obtained from Honeywell (Charlotte, NC, USA), while acetone, methanol, and ethanol were of analytical grade and were obtained from Química Moderna (Barueri, Brazil). All other materials were of pharmaceutical grade.

### 3.2. Methods

#### 3.2.1. Preparation of the Nanocapsule Suspensions

Licuri oil and avobenzone nanocapsule suspensions were prepared via interfacial deposition of the preformed polymer method [61], with modifications. An organic phase composed of Eudragit^®^ RS 100 (0.200 g), licuri oil (L, 0.300 g), avobenzone (A, 0.025 g), and acetone (50 mL) was stirred magnetically for 15 min at 37 °C. Afterward, the organic phase was injected into an aqueous phase (100 mL) containing polysorbate 80 (0.150 g). After 15 min of magnetic stirring, the suspension was concentrated on a rotary evaporator (Rotavapor^®^ RII, Büchi, Flawil, Switzerland) at 40 °C to remove acetone and part of the water, yielding a final volume of 10 mL. The resulting formulation containing 2.5 mg mL^−1^ avobenzone was designated NCL-A. For comparison purposes, polymeric nanocapsules without avobenzone (NCL) and polymeric nanocapsules prepared with medium-chain triglycerides (T) instead of licuri oil, with (NCT-A) and without avobenzone (NCT), were also prepared under the same experimental conditions. Additionally, to evaluate the influence of the polymeric layer, licuri oil nanoemulsions with (NEL-A) and without avobenzone (NEL) were prepared by omitting the polymer from the formulation (Table 3). All formulations were stored at room temperature, and those containing avobenzone were prepared in the dark.

#### 3.2.2. Characterization of the Nanocapsule Suspensions

The volume-weighted mean diameter (D[4,3]), particle size distribution, and span were determined via laser diffraction using a Mastersizer^®^ 2000 (Malvern Panalytical, Malvern, UK). The samples were added directly to the dispersion unit containing distilled water until an obscuration of 2–8% was achieved. A refractive index (RI) of 1.38 was used for Eudragit^®^ RS 100-based nanocapsules, whereas an RI of 1.44 was used for the licuri oil nanoemulsions [25].

The hydrodynamic particle diameter (*z*-average) and PDI of the formulations were determined by DLS using a ZetaSizer^®^ Nano ZS (Model ZEN 3600, Malvern Panalytical, Malvern, UK) after dilution (1:500, *v*/*v*) in ultrapure water. Measurements were performed at 25 °C using a detection angle of 173° NTA and a NanoSight LM10 instrument (Amesbury, UK). NCL-A and NCT-A were diluted (1:10,000, *v*/*v*) in ultrapure water and analyzed using a 638 nm red laser. The Brownian motion of individual particles was tracked frame-by-frame over a 60 s recording period and processed using device software (NTA 3.2 Built 126, Amesbury, UK).

Zeta potential was determined by electrophoretic mobility using a ZetaSizer^®^ Nano ZS (Model ZEN 3600, Malvern Panalytical, Malvern, UK) after dilution (1:500, *v*/*v*) in a 10 mmol L^−1^ aqueous sodium chloride solution. The pH was determined at room temperature by direct potentiometric measurement of the nanocapsule suspensions using a calibrated potentiometer (DM-22; Digimed, São Paulo, Brazil).

The morphology of NCL-A was evaluated by transmission electron microscopy (TEM; TECNAI G^2^ F20 operating at 200 kV) at Laboratório Central de Microscopia e Microanálise (PUCRS, Porto Alegre, Brazil). The nanocapsule suspension was diluted 1:10 (*v*/*v*) in ultrapure water, deposited onto a Formvar grid, and negatively stained with a 2% (*w*/*v*) aqueous uranyl acetate solution.

#### 3.2.3. HPLC Method for Avobenzone Quantification

Avobenzone quantification was performed according to the method described by Puglia et al. [59], with modifications. The chromatographic system consisted of a Luna^®^ C18 analytical column (150 mm × 4.5 mm, 5 μm; Phenomenex^®^, Torrance, CA, USA) protected by a guard column (SecurityGuard^TM^ C18, 4.0 mm × 3.0 mm; Phenomenex^®^, Torrance, CA, USA) coupled to an HPLC system equipped with a degasser (DGU-20A5), a solvent delivery system (LC-20AT), an autosampler (SIL-20A HT), a UV-Vis detector (SPD-20A), and a system controller (CBM-20A) (Shimadzu, Kyoto, Japan).

The mobile phase consisted of acetonitrile and water (90:10, *v*/*v*) with 1% acetic acid added to the aqueous phase. Chromatographic separation was performed at a flow rate of 1.0 mL min^−1^, using an injection volume of 30 μL and UV detection at 360 nm. The retention time for avobenzone was 5.26 min. The method was validated for specificity, linearity, accuracy, and precision (intraday, *n* = 6; interday, *n* = 9) according to official guidelines [62].

The method demonstrated specificity and linearity over the concentration range of 5.0–45 µg mL^−1^ (y = 195122x + 7609.7; r^2^ = 0.999). The accuracy was evaluated at low, medium, and high concentrations, with recovery values of 91.95%, 96.39%, and 97.66%, respectively, and RSD values less than 3.5%. Precision was evaluated using intraday and interday analyses, yielding RSD values of 2.20% and 3.20%, respectively. The limits of detection (LoD) and quantification (LoQ) were calculated using Equations (1) and (2), respectively:

(1) $$LD=3.3\cdot \sigma \cdot \;S^{-1}$$

(2) $$LQ=10\cdot \sigma \cdot \;S^{-1}$$

where σ is the standard deviation of the peak area response, and S is the slope of the calibration curve [62]. The LoD and LoQ values were 0.90 and 2.74 µg mL^−1^, respectively.

#### 3.2.4. Avobenzone Content and Encapsulation Efficiency

The avobenzone content (mg mL^−1^) in the formulations was determined after the samples (100 μL) were diluted in 10 mL of acetonitrile and manually shaken to ensure complete dissolution of all components. The samples were filtered through a 0.45 μm membrane filter (Millipore^®^) and analyzed according to the HPLC-UV method previously described.

Avobenzone encapsulation efficiency (EE%) was calculated based on the difference between the total avobenzone concentration and the nonencapsulated avobenzone concentration. The free avobenzone fraction was separated by ultrafiltration–centrifugation using an ultrafiltration device (10 kDa cutoff, Amicon^®^ Ultra, EMD Millipore, Billerica, MA, USA). Samples (NCL-A and NCT-A) were centrifuged at 1733× *g* for 10 min, and the resulting ultrafiltrates were analyzed by HPLC-UV according to the method previously described.

#### 3.2.5. Photostability Evaluations

The photostability study was carried out under simulated solar UV radiation using a mirrored chamber coupled with a UV light source (black light lamp F30W/T8/BL368, 95% UVA and 5% UVB, light intensity of 0.220 mW cm^−2^, corresponding to a total UVA radiant exposure of approximately 38.02 J cm^−2^ [63] over the 48 h assay period). Polystyrene cuvettes containing 1.7 mL of each sample were maintained under constant irradiation. Since this study aimed to evaluate the effect of the oily core on the photoprotective performance of the nanocapsules, only the nanocapsule formulations NCL-A and NCT-A were assessed. A free avobenzone solution prepared in acetonitrile and water (1:1, *v*/*v*) at the same avobenzone concentration as the nanocapsule formulations was also evaluated for comparison. The cumulative UVA radiant exposure was determined for each sampling time based on the constant irradiance of the UVA source (0.220 mW cm^−2^) and the accumulated exposure time. At predetermined time points (0, 15 min, 30 min, 1 h, 2 h, 4 h, 8 h, 12 h, 24 h, and 48 h), corresponding to cumulative UVA radiant exposures of 0, 0.20, 0.40, 0.79, 1.58, 3.17, 6.34, 9.50, 19.01, and 38.02 J cm^−2^, respectively, aliquots (100 μL) of each sample were collected, diluted in acetonitrile, and filtered through a 0.45 μm membrane (Millipore^®^) for avobenzone quantification by HPLC-UV according to the previously described methodology. For each formulation, the experimentally determined avobenzone content before the photostability assay (0 h) was defined as 100%, and the avobenzone content measured at each subsequent time point was expressed as a percentage relative to this initial value.

#### 3.2.6. In Vitro Antioxidant Assays

The antioxidant activity of the samples (NCL, NCL-A, NEL, NEL-A, NCT, NCT-A, licuri oil, and MCT) was evaluated using two radical scavenging assays: DPPH and ABTS.

##### DPPH Radical Scavenging Assay

The scavenging activity of 2,2-diphenyl-1-picrylhydrazyl (DPPH•) radicals was determined according to the method described by Gonzales et al. [64] with slight modifications. This assay is based on the ability of antioxidant compounds to donate hydrogen atoms to DPPH• radicals, thereby reducing them from the violet-colored form to the pale-yellow reduced form (DPPH-H).

A DPPH solution (20 mg mL^−1^) was prepared in methanol. For the assay, 3 mL of the DPPH solution was mixed with 1 mL of each sample or with methanol as the control. Licuri oil and MCT were also evaluated at the same concentration used in the formulations (30 mg mL^−1^). The mixtures were homogenized and kept in the dark for 2 h. Absorbance was measured at 515 nm using a UV-VIS spectrophotometer (T 80+, PG Instruments Limited, Leicestershire, UK), with methanol used as the blank. The percentage of deactivation was calculated according to Equation (3):

(3) $$\%\;deactivation=\;\frac{Ac-As}{Ac}\;\times \;100$$

where *Ac* is the absorbance of the control and *As* is the absorbance of the sample.

##### Deactivation of 2,2′-Azinobis-3-ethylbenzothiazoline-6-sulfonic Acid (ABTS) Radical

The antioxidant activity of the formulations was also evaluated using the ABTS radical cation (ABTS•^+^) decolorization assay, adapted from Re et al. [65]. The ABTS•^+^ radical was generated by reacting an aqueous ABTS solution (7 mmol L^−1^) with potassium persulfate (2.5 mmol mL^−1^), followed by incubation in the dark at room temperature for 12–16 h. The resulting radical solution was diluted in ethanol to obtain an absorbance of approximately 0.700 at 750 nm and equilibrated at 30 °C before analysis.

The samples were prepared by dissolving 0.2 mL of each formulation in 1.8 mL of ABTS working solution, and the absorbance was measured at 750 nm. Antioxidant activity was calculated as the percentage deactivation of ABTS according to Equation (4):

(4) $$\%\;deactivation=\;\frac{Ainicial\;-\;Afinal}{Ablank\;initial\;-\;Ablank\;final}\;\times \;100$$

where the blank consisted of ABTS solution without an antioxidant, and its deactivation percentage was used to correct the sample values.

#### 3.2.7. In Vitro Study of Irritation Potential Evaluation

The irritation potential of the nanocapsule suspensions and nanoemulsions (NCL, NCL-A, NEL, NEL-A, NCT, and NCT-A) was evaluated using the chorioallantoic membrane test (HET-CAM). Fertilized chicken eggs were incubated at 37.5 °C and 60% relative humidity and used on day 10 of incubation. The eggshell was carefully removed to expose the chorioallantoic membrane (CAM). Subsequently, 300 μL of each formulation (*n* = 5) was applied to the CAM, and the response was observed with the naked eye for 300 s to assess the potential irritant effect. A 0.9% NaCl solution served as the negative control, while a 1% sodium lauryl sulfate solution served as the positive control for vasoconstriction. A 0.1 mol L^−1^ NaOH solution was used as the positive control for hemorrhage and coagulation. The irritation score (IS) was calculated according to Equation (5) [46,66].

(5) $$IS=\frac{5\times \left(301-H\right)}{300}+\frac{7\times \left(301-V\right)}{300}+\frac{9\times (301-C)}{300}$$

where *H* is the hemorrhage time (s), *V* is the vasoconstriction time (s), and *C* is the coagulation time (s). The formulations were classified according to the irritability score as nonirritant (0–0.9), slight irritant (1.0–4.9), moderate irritant (5–8.9), or extreme irritant (9.0–21).

#### 3.2.8. Preparation of the Hydrogels

The hydrogels were prepared by adding 1.5% (*w*/*v*) hyaluronic acid (HA) or 2% (*w*/*v*) xanthan gum (XG) to nanocapsule suspensions containing licuri oil (NCL and NCL-A) supplemented with 0.25% (*w*/*v*) imidazolidinyl urea. The formulations were manually stirred until homogeneous formulations were obtained. After preparation, the formulations were refrigerated for 24 h with periodic homogenization to ensure complete polymer dispersion. Control formulations were also prepared using free avobenzone, water, NCT, NCT-A, NEL or NEL-A. The formulations were designed as HA-FA, HAH_2_O, HANCL, HANCL-A, HANCT, HANCT-A, HANEL, HANEL-A, XG-FA, XGH_2_O, XGNCL, XGNCL-A, XGNCT, XGNCT-A, XGNEL, and XGNEL-A (Table 4).

#### 3.2.9. Characterization of the Hydrogels

The formulations were characterized by mean particle diameter, polydispersity index, pH, avobenzone content, and rheological behavior. The mean hydrodynamic diameter (*z*-average) and the polydispersity index (PDI) were evaluated by dynamic light scattering using a ZetaSizer^®^ Nano ZS (Malvern Panalytical, Malvern, UK). The formulations were diluted (1:500, *v*/*v*) in ultrapure water. The pH was determined at room temperature using a previously calibrated digital potentiometer (DM-22, Digimed, São Paulo, Brazil) by direct immersion of the electrode into the diluted formulations. For a 10% (*w*/*v*) dilution, 1 g of the formulation was dispersed in 9 mL of ultrapure water.

To determine the avobenzone content in the hydrogels, 100 mg of each formulation was placed in a volumetric flask, and compound extraction was performed by adding acetonitrile and using an ultrasound bath (5 h). Afterward, the samples were filtered through a 0.45 μm membrane (Millipore^®^), and avobenzone quantification was performed by HPLC-UV using a previously described methodology.

#### 3.2.10. Rheological Behavior of Hydrogels

Rheological measurements were performed using a rheometer equipped with parallel plate geometry (25 mm diameter) and a 0.5 mm gap. Measurements were carried out at 25 ± 1 °C in a thermostatically controlled water bath. During analysis, the shear rate was increased from 0 to 300 s^−1^ (ascending curve) and subsequently decreased from 300 to 0 s^−1^ (descending curve). The rheological profile and thixotropic behavior of the formulations were evaluated based on the hysteresis area between the ascending and descending flow curves. Rheological analyses were performed at the Laboratório de Biomateriais Poliméricos (Poli-Bio) at UFRGS.

#### 3.2.11. Evaluation of the UV Light Absorption/Scatter Properties of the Hydrogels

The hydrogels (HAH_2_O, HANCL, HANCL-A, HANCT, HANCT-A, XGH_2_O, XGNCL, XGNCL-A, XGNCT, and XGNCT-A) were scanned in the UV range from 280 to 400 nm using a UV spectrophotometer. Additionally, separate formulations containing free avobenzone dispersed in polysorbate 80 were prepared and then incorporated into either hyaluronic acid (HA-FA) or xanthan gum (XG-FA). Each formulation was transferred into a quartz cuvette with a capacity of 2.6 μL and an optical path length of 0.01 mm (106-QS; Hellma, Müllheim, Germany) and sealed with a holder (010.000; Hellma, Müllheim, Germany). The samples were scanned in the UV range from 280 to 400 nm.

#### 3.2.12. In Vitro Evaluation of Avobenzone Skin Penetration and Permeation

Permeability studies were performed using an automated Franz cell diffusion cell system (MicroettePlus Multi-Group^®^, Hanson Research Corporation, Chatsworth, CA, USA) with porcine ear skin as the membrane. Hydrogels containing avobenzone-loaded polymeric nanocapsules and avobenzone-loaded nanoemulsions (HANCL-A, HANCT-A, HANEL-A, XGNCL-A, XGNCT-A, and XGNEL-A) were weighed (200 mg) and applied to the skin surface area (1.76 cm^2^). The receptor compartment was filled with 7 mL of phosphate-buffered solution (pH 7.4) containing 4% (*w*/*v*) polysorbate 80 to maintain sink conditions. The receptor medium was maintained at 32.5 ± 2 °C and continuously stirred using a magnetic stir bar. Aliquots (1 mL) were automatically collected from the receptor medium at 30 min and at 1, 2, 4, 6, and 8 h after application, and the same volume of fresh receptor solution was replaced. The samples were assayed by HPLC-UV according to the previously described methodology.

##### Skin Layer Separation Procedure

Tape stripping is a widely used method for assessing the penetration profile of topical formulations by sequentially removing stratum corneum layers, thereby quantifying the compounds retained within the stratum corneum [67]. In the present study, this technique was used to separate the stratum corneum from viable skin, thereby enabling quantification of avobenzone retained in each skin compartment. After 8 h of permeation, the residual hydrogel was gently removed from the skin surface using cotton. Subsequently, 18 adhesive tape strips (3 M Scotch^®^, Porto Alegre, Brazil) were sequentially applied to each membrane to remove the stratum corneum. After tape stripping, the remaining skin tissue (viable skin) was cut into small fragments. The tape strips and viable skin samples were transferred to separate Falcon tubes containing acetonitrile (5 mL), vortexed for 1 min, and sonicated for 30 min at 40 °C. The samples were filtered through a 0.45 μm membrane filter (Millipore^®^) and analyzed by HPLC-UV according to the previously described methodology.

Owing to the low analyte concentrations expected in the skin permeation samples, a new calibration curve was established using a lower concentration range. The curve was linear over the concentration range of 0.1–75 μg mL^−1^ (r^2^ = 0.998), with limits of detection and quantification of 0.05 and 0.15 μg mL^−1^, respectively.

#### 3.2.13. Statistical Analysis

The results are expressed as the mean ± standard deviation. Statistical analysis was performed using GraphPad Prism software (Version 8.0, GraphPad Software, San Diego, CA, USA), and the statistical significance between groups was evaluated by one-way analysis of variance (ANOVA), followed by Tukey’s multiple comparison test (*p* ≤ 0.05).

## 4. Conclusions

This study demonstrated that licuri oil is a suitable natural alternative to medium-chain triglycerides as the oily core of cationic polymeric nanocapsules intended for topical formulations containing encapsulated avobenzone. The licuri oil-based nanocapsules, similar to the MCT-based nanocapsules, exhibited a nanometric size, narrow size distribution, positive zeta potential, and an avobenzone encapsulation efficiency above 98%. Most notably, compared with the free UV filter, the licuri oil-based nanocapsules provided a photostabilizing effect on avobenzone equivalent to that obtained with MCT. In addition to matching MCT performance, licuri oil contributed additional antioxidant activity to the formulations and showed low HET-CAM irritation potential. The developed nanostructured systems were successfully incorporated into hydrogels with suitable physicochemical and rheological properties for topical application, enhancing UV absorption, and modulating the cutaneous distribution of avobenzone, with no detectable avobenzone in the receptor medium throughout the 8 h in vitro study under the tested conditions.

## Figures and Tables

**Table 1:** Physicochemical characteristics of developed nanoformulations formulations.

Formulation	D[4,3] (nm)	Span	*z*-Average (nm)	PDI	Zeta Potential (mV)	pH
NCL	140 ± 2 ^a^	1.22 ± 0.01 ^ac^	146 ± 7 ^ab^	0.13 ± 0.01	+12.80 ± 1.14 ^a^	4.6 ± 0.1 ^a^
NCL-A	158 ± 8 ^a^	1.37 ± 0.15 ^ab^	164 ± 14 ^b^	0.15 ± 0.02	+11.03 ± 0.75 ^a^	4.8 ± 0.1 ^a^
NCT	128 ± 1 ^a^	0.88 ± 0.04 ^d^	131 ± 1 ^a^	0.10 ± 0.08	+12.00 ± 2.50 ^a^	3.6 ± 0.1 ^bc^
NCT-A	125 ± 1 ^a^	0.92 ± 0.08 ^cd^	131 ± 1 ^a^	0.11 ± 0.06	+11.63 ± 3.01 ^a^	3.9 ± 0.2 ^b^
NEL	279 ± 17 ^b^	1.47 ± 0.11 ^ab^	205 ± 5 ^c^	0.15 ± 0.01	−8.45 ± 0.62 ^b^	3.7 ± 0.1 ^b^
NEL-A	288 ± 97 ^b^	1.60 ± 0.20 ^b^	209 ± 10 ^c^	0.16 ± 0.01	−7.66 ± 0.78 ^b^	3.4 ± 0.2 ^c^

The data are expressed as the mean ± standard deviation (*n* = 3). D[4,3]: volume-weighted mean diameter determined; PDI: polydispersity index. Different letters within the same column indicate statistically significant differences between formulations (*p* ≤ 0.05), as determined by one-way ANOVA followed by Tukey’s test. NCL: polymeric nanocapsule with licuri oil; NCL-A: polymeric nanocapsule with licuri oil and avobenzone; NCT: polymeric nanocapsule with medium-chain triglycerides; NCT-A: polymeric nanocapsule with medium-chain triglycerides and avobenzone; NEL: nanoemulsion with licuri oil; NEL-A: nanoemulsion with licuri oil and avobenzone.

**Table 2:** Physicochemical characteristics of developed hydrogels.

Formulation	*z*-Average (nm)	PDI	pH	Avobenzone Content (%)
HANCL	168 ± 3 ^cd^	0.27 ± 0.01 ^ab^	5.53 ± 0.04 ^a^	-
HANCL-A	156 ± 6 ^c^	0.24 ± 0.03 ^a^	6.00 ± 0.12 ^b^	104.16 ± 0.80 ^a^
HANEL	188 ± 4 ^e^	0.22 ± 0.02 ^a^	5.64 ± 0.05 ^a^	-
HANEL-A	204 ± 3 ^f^	0.26 ± 0.01 ^a^	5.95 ± 0.19 ^b^	107.47 ± 2.83 ^a^
XGNCL	137 ± 2 ^b^	0.26 ± 0.01 ^a^	6.07 ± 0.04 ^b^	-
XGNCL-A	117 ± 2 ^a^	0.26 ± 0.01 ^a^	6.15 ± 0.09 ^b^	94.11 ± 0.99 ^b^
XGNEL	176 ± 6 ^de^	0.19 ± 0.06 ^ab^	6.09 ± 0.11 ^b^	-
XGNEL-A	189 ± 9 ^e^	0.17 ± 0.03 ^b^	6.05 ± 0.07 ^b^	87.33 ± 2.38 ^c^

The values are expressed as the mean ± standard deviation (*n* = 3). PDI (polydispersity index). Different letters within the same column indicate statistically significant differences between formulations (*p* ≤ 0.05), as determined by one-way ANOVA followed by Tukey’s test. HANCL: hyaluronic acid hydrogel containing polymeric nanocapsules with licuri oil; HANCL-A: hyaluronic acid hydrogel containing polymeric nanocapsules with licuri oil and avobenzone; HANEL: hyaluronic acid hydrogel containing nanoemulsion with licuri oil; HANEL-A: hyaluronic acid hydrogel containing nanoemulsion with licuri oil and avobenzone; XGNCL: xanthan gum hydrogel containing polymeric nanocapsules with licuri oil; XGNCL-A: xanthan gum hydrogel containing polymeric nanocapsules with licuri oil and avobenzone; XGNEL: xanthan gum hydrogel containing nanoemulsion with licuri oil; XGNEL-A: xanthan gum hydrogel containing nanoemulsion with licuri oil and avobenzone.

**Table 3:** Composition of polymeric nanocapsules.

Formulation	NCL	NCL-A	NCT	NCT-A	NEL	NEL-A
Organic phase						
Eudragit^®^ RS100	0.200 g	0.200 g	0.200 g	0.200 g	-	-
Licuri oil	0.300 g	0.300 g	-	-	0.300 g	0.300 g
MCT	-	-	0.300 g	0.300 g	-	-
Acetone	50 mL	50 mL	50 mL	50 mL	50 mL	50 mL
Avobenzone	-	0.025 g	-	0.025 g	-	0.025 g
Aqueous phase						
Polysorbate 80	0.150 g	0.150 g	0.150 g	0.150 g	0.150 g	0.150 g
Water	100 mL	100 mL	100 mL	100 mL	100 mL	100 mL

**Table 4:** Composition of hydrogels.

Formulations	Components										
	HA	XG	Imidazolidinyl Urea	FA	H_2_O	NCL	NCL-A	NCT	NCT-A	NEL	NEL-A
HA-FA	0.150 g	-	0.025 g	0.025 g	10 mL	-	-	-	-	-	-
HAH_2_O	0.150 g	-	0.025 g	-	10 mL	-	-	-	-	-	-
HANCL	0.150 g	-	0.025 g	-	-	10 mL	-	-	-	-	-
HANCL-A	0.150 g	-	0.025 g	-	-	-	10 mL	-	-	-	-
HANCT	0.150 g	-	0.025 g	-	-	-	-	10 mL	-	-	-
HANCT-A	0.150 g	-	0.025 g	-	-	-	-	-	10 mL	-	-
HANEL	0.150 g	-	0.025 g	-	-	-	-	-	-	10 mL	-
HANEL-A	0.150 g	-	0.025 g	-	-	-	-	-	-	-	10 mL
XG-FA	-	0.200 g	0.025 g	0.025 g	10 mL	-	-	-	-	-	-
XGH_2_O	-	0.200 g	0.025 g	-	10 mL	-	-	-	-	-	-
XGNCL	-	0.200 g	0.025 g	-	-	10 mL	-	-	-	-	-
XGNCL-A	-	0.200 g	0.025 g	-	-	-	10 mL	-	-	-	-
XGNCT	-	0.200 g	0.025 g	-	-	-	-	10 mL	-	-	-
XGNCT-A	-	0.200 g	0.025 g	-	-	-	-	-	10 mL	-	-
XGNEL	-	0.200 g	0.025 g	-	-	-	-	-		10 mL	-
XGNEL-A	-	0.200 g	0.025 g	-	-	-	-	-		-	10 mL

HA: hyaluronic acid; XG: xanthan gum; FA: free avobenzone; NCL: polymeric nanocapsule with licuri oil; NCL-A: polymeric nanocapsule with licuri oil and avobenzone; NCT: polymeric nanocapsule with medium-chain triglycerides; NCT-A: polymeric nanocapsule with medium-chain triglycerides and avobenzone; NEL: nanoemulsion with licuri oil; NEL-A: nanoemulsion with licuri oil and avobenzone.

## Data Availability

The data presented in this study are available on request from the corresponding author.

## Abbreviations

The following abbreviations are used in this manuscript:

A	Avobenzone
ANOVA	Analysis of variance
DLS	Dynamic light scattering
EE (%)	Encapsulation efficiency
FA	Free avobenzone
HA	Hyaluronic acid
HA-FA	Hyaluronic acid hydrogel with free avobenzone
HAH_2_O	Hyaluronic acid hydrogel with water
HANCL	Hyaluronic acid hydrogel containing polymeric nanocapsules with licuri oil
HANCL-A	Hyaluronic acid hydrogel containing polymeric nanocapsules with licuri oil and avobenzone
HANCT	Hyaluronic acid hydrogel containing polymeric nanocapsules with medium-chain triglycerides
HANCT-A	Hyaluronic acid hydrogel containing polymeric nanocapsules with medium-chain triglycerides and avobenzone
HANEL	Hyaluronic acid hydrogel containing nanoemulsion with licuri oil
HANEL-A	Hyaluronic acid hydrogel containing nanoemulsion with licuri oil and avobenzone
HET-CAM	Hen’s Egg Test–Chorioallantoic Membrane
HPLC-UV	High-performance liquid chromatography with UV detection
ICH	International Council for Harmonisation
IS	Irritation score
LoD	Limit of detection
LoQ	Limit of quantification
MCT	Medium-chain triglycerides
NCL	Polymeric nanocapsules with licuri oil
NCL-A	Polymeric nanocapsules with licuri oil and avobenzone
NCT	Polymeric nanocapsules with medium-chain triglycerides
NCT-A	Polymeric nanocapsules with medium-chain triglycerides and avobenzone
NEL	Nanoemulsion with licuri oil
NEL-A	Nanoemulsion with licuri oil and avobenzone
NTA	Nanoparticle tracking analysis
PDI	Polydispersity index
RI	Refractive index
ROS	Reactive oxygen species
SPF	Solar protection factor
TEM	Transmission electron microscopy
UV	Ultraviolet
UVA	Ultraviolet A
UVB	Ultraviolet B
XG	Xanthan gum
XG-FA	Xanthan gum hydrogel with free avobenzone
XGH_2_O	Xanthan gum hydrogel with water
XGNCL	Xanthan gum hydrogel containing polymeric nanocapsules with licuri oil
XGNCL-A	Xanthan gum hydrogel containing polymeric nanocapsules with licuri oil and avobenzone
XGNCT	Xanthan gum hydrogel containing polymeric nanocapsules with medium-chain triglycerides
XGNCT-A	Xanthan gum hydrogel containing polymeric nanocapsules with medium-chain triglycerides and avobenzone
XGNEL	Xanthan gum hydrogel containing nanoemulsion with licuri oil
XGNEL-A	Xanthan gum hydrogel containing nanoemulsion with licuri oil and avobenzone
